# Specialty Grand Challenge for Molecular Signalling and Pathways in Molecular Neuroscience

**DOI:** 10.3389/fnmol.2021.694776

**Published:** 2021-05-24

**Authors:** Jean-Marc Taymans

**Affiliations:** Univ. Lille, Inserm, CHU Lille, UMR-S 1172 - LilNCog - Lille Neuroscience & Cognition, Lille, France

**Keywords:** chemical biology, pharmacology, molecular manipulation, network, omic analyses, synapse, protein complex, upstream/downstream process

It can be argued that it has never been more exciting than right now to be researching Molecular Signalling and Pathways in Neuroscience. This is the study devoted to identifying molecular and cellular players that underlie the structure, design and function of the brain across all levels. This study is not new and evolved with the major discoveries of biological sciences, including those discoveries that led to our fundamental understanding that genetic information is encoded by DNA, that is transcribed to RNA, that is translated to proteins, that in turn organise themselves together with other cellular biomolecules to carry out fundamental tasks of the cell. It is important to point out that signalling is a concept that existed prior to the discovery of DNA's genetic code. Indeed, work from the study of hormones had already pointed to the notion that processes within the cell could be influenced by the binding of ligands to cell surface receptors and this could then lead to important changes in tissues and organisms. Perhaps one of the most emblematic examples of such discoveries in neuroscience is the report in 1951 of a factor in tumour extracts that can cause neurite outgrowth and that was therefore aptly called nerve growth factor (Levi-Montalcini and Hamburger, 1951). Other discoveries, such as the effect of morphine, caffeine or even the effects of reward on the brain and behaviour were also known long before the precise genes and sequences were identified of the receptors involved, i.e., opioid, adrenergic and dopamine receptors, respectively.

One can only marvel at these accomplishments in Molecular Signalling and Pathways in Neuroscience done in the pre-genomic era. How envious these researchers would be of us and all of the resources we have at our disposal today. For instance, we have access to such fundamental knowledge as genome sequences (for humans as well as for multiple other species including many that are used as model organisms) (Lander et al., 2001; Venter et al., 2001), databases with tissue-specific gene expression patterns (GTEx, Protein Atlas) (GTEx Consortium, 2013; Uhlén et al., 2015), databases with key post-translational modifications on proteins to name just a few. Technologies today also enable studies that would not have been possible in the pre-genomic age. Who can deny the spectacular advances in our genomic age of “omics” technologies, advances in culturing or transgenesis techniques or advances in imaging or genome editing? We truly have an enormous capacity today to answer questions in Molecular Signalling and Pathways in Neuroscience. Questions such as: What are key molecular switches that govern the activity of brain signalling pathways? How are these linked to neuronal phenotypes in experimental model systems and in humans? We are ideally positioned to address such questions, such that should we be confronted with a specific brain protein that we want to know everything about, there is no doubt that by focusing our attention, creativity and resources to that protein, we can fully expect to have a good idea of that protein's role in the brain within a few years.

Does this mean that today we somehow have it “easy” in researching Molecular Signalling and Pathways in Neuroscience given the wealth of knowledge and technologies we benefit from? Not at all, because the task ahead is gargantuan. This can be illustrated by the elucidation of the human genome itself. When first published, the advance was so monumentous that it seemed that unlocking the mysteries of gene functions would be only a matter of time. Now, 2 decades along, we know that our work on elucidating gene function in general and in the brain specifically is far from over. For instance, despite having fully sequenced the human genome, the precise number of genes in our genome remains to be determined (Salzberg, 2018; Willyard, 2018). As far as the study of brain signalling cascades goes, an overview of the complexity has emerged with for instance about 13,000 brain proteins identified (Sharma et al., 2015). While data and literature mining has allowed for canonical pathways to be defined, it is important to bear in mind that many proteins remain to be individually examined for their role in signalling in the brain. It is important for scientists to pursue the signalling work, not only by confirming canonical pathways, but also by identifying novel pathways, establishing links and cross-talk between pathways and refining the intricacies of functioning of known pathways. Our grand challenge consists therefore not only to build on known canonical pathways but also to uncover new and as yet unknown pathways.

Examples of the experimental approaches pertinent to this section are schematically represented in Figure 1. A few of these approaches concern individual focus proteins that can be characterised in depth to gain knowledge on signalling pathways. This may include studying the biochemical and structural properties of this signalling protein, to understand whether there are properties that can lead to this protein's activation or inactivation, such as post-translational modifications, di- or multimerisation, nucleotide binding, enzymatic activities etc. Such work may involve biochemical approaches or also biomolecular modelling. Of course, being integrated in a pathway suggests that there are physical interactions to be identified and characterised. Interactomics screening technologies are available to identify binding partners of focus proteins such as yeast 2 hybrid screening, phage display, mass spectrometric analysis of protein complexes isolated by affinity purification under native conditions, to name a few. As in any screening procedure, identified interactors are tested with additional binding techniques in order to confirm and characterise the binding, up to determining binding affinities. In characterising interactions within a signalling cascade, an important aspect is to determine whether interactions are modulated in different conditions. For instance, is the interaction dependent on certain post-translational modifications or on specific protein conformations? Such information provides a first molecular basis for modulation of the signalling pathway. Besides direct interactors, there are other partners of signalling proteins than can be discerned in a signalling cascade, such as upstream regulators and downstream partners. Upstream regulators can be specifically screened for, especially if there is a clear activation/inactivation event that can be measured such as a post-translational modification or conformational change, using reverse genetics or chemical biology screens. For instance, to search for regulators of a phosphorylation event, an siRNA or pharmacological screen can be performed with an siRNA library or compound libraries directed against kinases. Similarly, downstream partners such as substrates or effectors can be identified. Using again the example of phosphorylation, phosphoproteomics screening can be performed in conditions of kinase knockouts or inhibition. In both types of studies, upstream regulators or downstream partners can be confirmed and characterised with additional focused testing. Note that the examples given here in no way constitute an exhaustive list of approaches to identify steps of molecular pathways in neuroscience. Part of this grand challenge to study pathways in neuroscience is to do so not only with existing approaches but also to exploit the wealth of novel technological opportunities that would allow to go deeper into discovering and characterising signalling steps.

**Figure 1:**
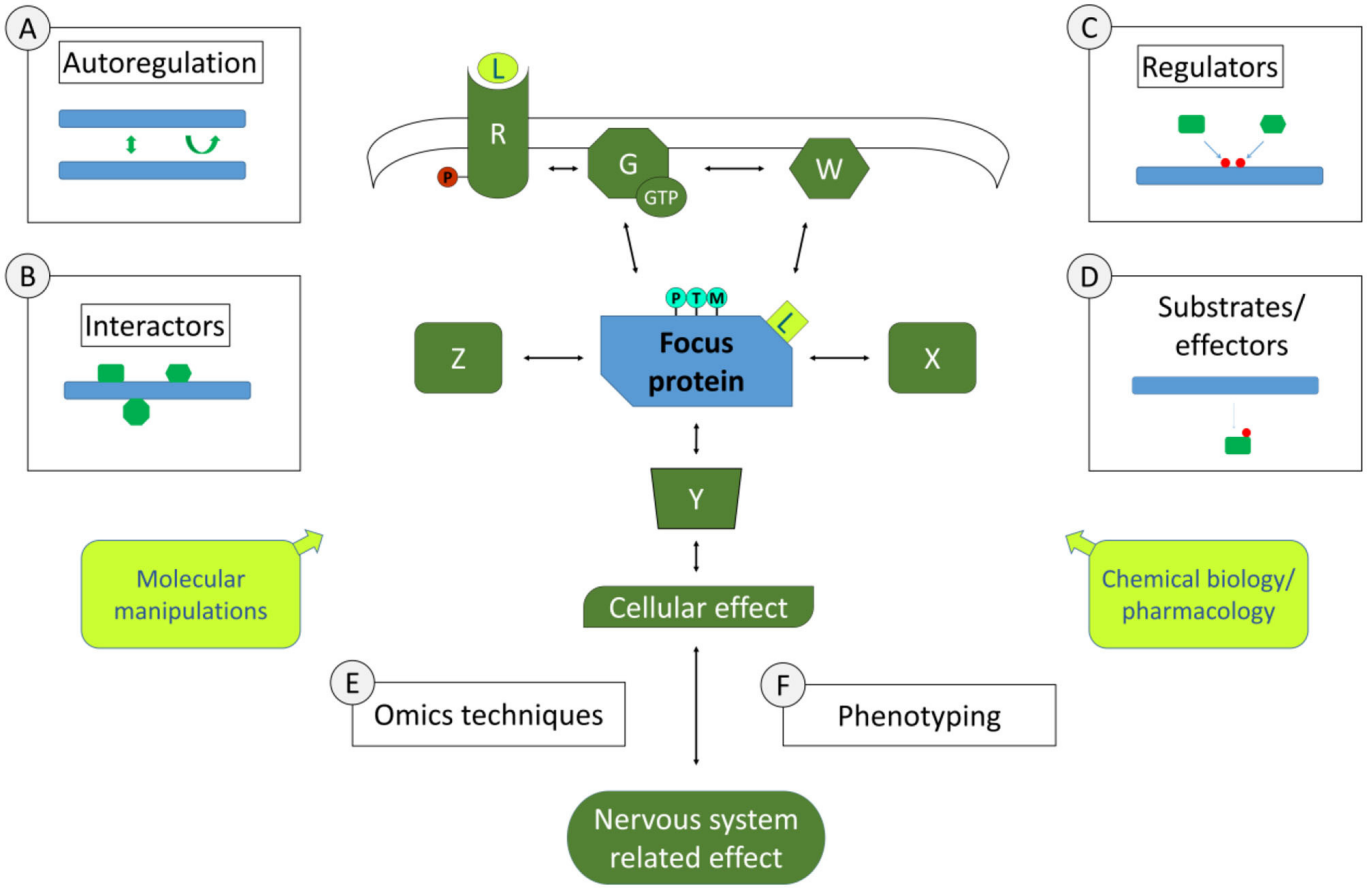
Molecular Signalling and Pathways in Neuroscience. Depicted centrally is a simplified schematic of elements of a signalling pathway involved in nervous system functions. Molecular signalling pathways are of interest in their whole (all elements in green and the one in blue) as well as in the characteristics of individual components (symbolised by the blue focus protein). Study around focus proteins can begin to inform in the pathway involved by the study of its autoregulation (approach A, if applicable), its direct physical interactors (approach B, symbolised by theoretical interactors such as X, Z), its upstream regulators [approach C, with theoretical regulators W, or G (G-proteins) and R (receptors), leading also potentially to post-translational modifications (PTMs)], its downstream substrates (Y). Using Omics approaches (E) such as proteomics, transcriptomics etc. as a way to define the profile of cells or tissues in specific conditions, it is also possible to make deductions on the pathway that yielded the measured Omics profile. Omics profiles are also a potential phenotype (F) in their own right, as are more specific measures of the cells involved in the nervous systems related effects, including how this affects specific cellular functions or organelles and how this impacts cell-cell interactions, neuronal circuits or functions of specific tissues. In Molecular Neuroscience, we also have a myriad of tools/approaches to explore pathways functions (yellow boxes), including molecular manipulations such as knock-out, knock-down, knock-in (including via genome-editing techniques such as CRISPR/CAS), protein-protein interaction modulators as well as chemical biology or pharmacological maniplulations such as synthetic or natural small molecule or peptide ligands (L). The ultimate goal is to gain a full picture of the pathway under study, how it is linked to nervous system functions and the equilibria that exist within the pathways and in relation to other pathways under normal and pathological conditions.

Besides individual protein focused approaches, other approaches in elucidating signalling pathways are based on transcriptome-wide, proteome-wide or similar Omics approaches. Screening technologies to identify pathway activity and regulation on a broad scale have become standard tools, such as sequencing technologies or proteomics techniques (including post-transcriptional or post-translational modifications). Screening results, or Omics profiles, can be used for *in silico* pathway analysis to make deductions on the pathway that is modified in specific conditions, including extrapolating signalling patterns by analysing effects on expression profiles of genes, proteins, post-transcriptional/translational modifications of cells or tissues in specific conditions. These bioinformatics approaches are particularly useful to point to pathways or processes that are not necessarily identified in individual focus protein approaches. In addition, Omics approaches also shed light on the processes directly involved in gene expression patterns that can also be studied in their own right such as the biology of transcription factors, epigenetics or post-transcriptional modifications such as RNA splicing and editing.

Dissecting out the intricacies of signalling pathways at the molecular level is fascinating in its own right; however, it becomes downright exciting when pathways are linked to the functioning of the nervous system in health and disease, with a particular focus on synaptic and cellular proteins that define cellular identities. This is of course the element that gives the Molecular Signalling and Pathways in Neuroscience challenge all of its purpose. How do individual steps in signalling pathways lead to functional effects in cells and organisms? In many cases, clues will have arisen from molecular studies as to which phenotypic effects may be expected from a specific signalling cascade. Alternatively, phenotypic screening will be required to determine suitable phenotypes to test in relation to a specific signalling pathway. In either case, a multitude of molecular and pharmacological approaches are at our disposal to explore the pathway-phenotype relationships. Individual proteins can be modified in model systems (cellular, multicellular, and organismal), including expression of mutant forms of the proteins (exogenous expression or knock-in models) or gene knock-outs or knock-downs, and the effect of this modification measured on specific phenotypes, for example how the knock-out of a synaptic protein would affect synaptic vesicle release or neurite complexity in primary neurons. In addition, pharmacological approaches exist, for instance when tool compounds, existing or newly developed, are used to activate or inactivate a protein within the signalling cascade, when it is again possible to explore how this modification modifies the pathway's phenotypes. These few examples given here are again not limitative relative to our challenge. The activity of linking pathways to phenotypes is in continuous progression both at the scientific and at the technological level and this should be reflected in the submissions to this section. The pathway-phenotype relationships are much more than the icing on the cake of Molecular Signalling and Pathways in Neuroscience. It is precisely this relationship that allows us to go further both for fundamental studies and for applied research. Indeed, it allows further dissection of pathways, such as uncharacterized partners within these pathways, specific post-translational modifications within pathways or the phenotypes of pathways in specific conditions. It also allows development of tools to modulate pathway phenotypes such as developing pharmacological agents targeting specific elements of pathways such as key enzymes, receptors or protein-protein interactions. Linked to this, the understanding of pathway-phenotype relationships allow the possibility to develop models for this pathway that can again have fundamental scientific goals or destined for specific applications such as disease modelling.

As the old saying goes, we have no idea what the future has in store for us. However, of one thing we can be certain: we have never been in a better position to make leaping advances in Molecular Neuroscience as today. With amazing resources available to us such as whole genomes and proteomes of multiple species, nervous system expression patterns of just about any gene, a broad and expanding range of techniques for molecular manipulation of cells and model organisms, idem for pharmacological tools, a broad and expanding range of technologies to characterise cellular and organismal phenotypes, it is both our duty and our privilege to address the challenge of elucidating Molecular Signalling and Pathways in Neuroscience. No doubt that scientists will be hard at work to make this happen and we can look forward in hopeful anticipation to many exciting studies to come in this field.

## Author Contributions

J-MT: wrote the manuscript.

## Conflict of Interest

The author declares that the research was conducted in the absence of any commercial or financial relationships that could be construed as a potential conflict of interest.
